# The Shared Pleasure Paradigm: A study in an observational birth cohort in South Africa

**DOI:** 10.1007/s00737-021-01199-0

**Published:** 2022-01-05

**Authors:** Anusha Lachman, Esme R. Jordaan, Micky Stern, Kirsten A. Donald, Nadia Hoffman, Marilyn T. Lake, Heather J. Zar, Dana J. H. Niehaus, Kaija Puura, Dan J. Stein

**Affiliations:** 1 Department of Psychiatry, Stellenbosch University, Cape Town, South Africa; 2 Biostatistics Unit, South African Medical Research Council, Parow, South Africa; 3 Department of Statistics and Population Studies, University of the Western Cape, Cape Town, South Africa; 4 South African Medical Research Council Unit On Risk and Resilience in Mental Disorders, Cape Town, South Africa; 5 Department of Psychiatry and Neuroscience Institute, University of Cape Town, Cape Town, South Africa; 6 Department of Paediatrics and Child Health, Red Cross War Memorial Children’s Hospital, University of Cape Town, Cape Town, South Africa; 7 South African Medical Research Council Unit On Child and Adolescent Health, Cape Town, South Africa; 8 Department of Child Psychiatry, Tampere University and Tampere University Hospital, Tampere, Finland

**Keywords:** Shared pleasure, Synchronicity, Mother–infant relationship, Infant development, Low and middle-income country 35

## Abstract

Mother–infant dyads in low- and middle-income countries (LMICs) may be exposed to a range of factors associated with suboptimal development. Optimal infant development is likely supported by synchronicity in the early mother–infant relationship, but limited corroborative research is available in LMICs. The Drakenstein Child Health Study (DCHS) provided an opportunity to study this synchronicity and its associations in South Africa. A South African birth cohort study investigating early-life determinants of child health in a LMIC context provided participants. The Shared Pleasure (SP) paradigm helped assess early mother–infant synchronicity in videos of a sub-set of 291 mother–infant dyads at their 14-week well baby visit. General linear regression models investigated the relationship between selected maternal and infant characteristics and the presence of Shared Pleasure moments. Out of a possible 291 dyads, 82% (n = 239) yielded Shared Pleasure moments. The mean age of mothers was 27 years, while infant sex distribution comprised 54% females and 46% males. The shortest single Shared Pleasure moment lasted at least 0.5 s and the longest 28 s. Shared Pleasure moments were associated with higher gestation age at delivery (*p* = 0.008) and higher infant birth weight (*p* = 0.006), but were not related to mother's mental health and infant health outcomes at 14 weeks. The high frequency of positive Shared Pleasure moments in reciprocal dyadic interactions in this sample suggests that significant disruption in shared pleasure may be present only in extreme cases (e.g. mothers with severe mental disorders). Further work is needed to investigate the mechanisms underlying the associations between early mother–infant synchronicity and better outcomes noted here, and to assess whether SP may serve as a culturally appropriate screen for assessing connectedness.

**Supplementary Information:**

The online version contains supplementary material available at 10.1007/s00737-021-01199-0.

## Introduction

Mother–infant dyads in low- and middle-income countries (LMICs) may be exposed to a range of factors associated with suboptimal developmental outcomes. Substantial numbers of perinatal mental health disorders occur in LMICs, particularly in poorer peri-urban and rural areas (Fisher et al. 2012; Brittain et al. 2015). The early social environment fundamentally determines early child development and influences long-term child health outcomes (Maggi et al. 2010; Richter et al. 2020). Risk factors such as socio-economic stressors, poor social support and substance use, perinatal mental health disorders may impact and undermine mothers’ capacity to respond optimally to infants’ needs (WHO 2018). Adverse conditions like poverty inhibit parents’ capacity for providing security and assuring good developmental outcomes, especially for mothers vulnerable to mental health disorders (Patel et al. 2008). Early parent–infant relationships are key contributors to the socio-emotional development of growing children (Wachs et al. 2009; Richter et al. 2017).

Synchronicity within early mother–infant relationships seemingly supports optimal infant development, but there is limited relevant research in LMICs (Leclère et al. 2014). Synchronised behaviours like mutual gaze and gaze following between mothers and infants allegedly create the foundation of early social connectedness and regulation (Brooks and Meltzoff 2013), with positive emotions shared in meaningful relationships contributing social, intellectual and psychological resources (Ramsey and Gentzler 2015). Sharing positive affect in parent–infant interactions fuels the organisation of early infant experiences of socialisation (Feldman 2007), and positive caregiver–child interactions help infants develop socially and emotionally. Demonstrations of affection convey empathy to infants, manage responses to their dependence and model regulatory behaviours, and positive emotional interactions consistently increase mothers’ influence on and connectedness to their infants (Santamaria et al. 2020). The WHO’s Nurturing Care Framework (2018) highlights bidirectional communication and enjoyable, stimulating care as core to the provision of responsive care within healthy mother–child relationships.

Few studies have explored mother–infant relationships and synchronicity in LMICs (Donald et al. 2019) and few tools for assessing synchronicity have been validated in LMICs (Keller et al. 2018). Most studies in this setting focused on negative infant–caregiver interactions (Murray et al. 1996; Cooper et al. 2002; Christodoulou et al. 2019) with limited work exploring “Shared Pleasure (SP)” (Lachman et al. 2021). The former studies in similar settings in South Africa, reported more negative engagements (less sensitivity, less positive affect, less talking) between depressed mothers and their children and interventions were targeted at identifying and improving these negative interactions. Shared Pleasure is ideally measured in younger infants. During the first two months of life, touch gives way to gaze as a mode of interpersonal relatedness and gaze synchrony becomes the main vehicle for social interactions. (Feldman et al. 1999; Feldman 2007). By three months of age, an infant is seen to be more communicative and socially focused. This includes the emergence of emotions like sadness and joy, as well as social smiling. Younger infants are more likely to engage in direct face interactions as compared to older infants who are able to crawl away, thus limiting spontaneous opportunities for eye contact and positive emotional expressions (Sallquist et al. 2010; Puura et al. 2019).Two months seem to be the age at which mothers smile most automatically at their infants (Puura et al. 2019).

Culture influences a mothers’ behaviour towards her infant, and different communitiesmay emphasise different dimensions of interactions with their children. At the same time, it is problematic to assert that all mothers belonging to a particular cultural community behave in the same way. Parenting behaviours may be influenced by a broad range of determinants, including psychological and societal factors. At the same time, reciprocal positive SP interactions involve innate mechanisms, may overlap across different cultures, and can be measured effectively through direct observation in a range of settings.

The Drakenstein Child Health Study (DCHS), an ongoing birth cohort study in South Africa, assesses a range of maternal and infant measures (Stein et al. 2015; Zar et al. 2015). The current study aimed to explore the prevalence of shared pleasure moments in mother–infant dyads, to investigate associations of maternal and infact characteristics with the frequency and occurrence of shared pleasure. We hypothesised that greater SP would be associated with better maternal mental health and early childhood development.

## Methods

### Site

The Drakenstein Child Health Study (DCHS) is a population-based birth cohort study investigating early-life determinants of child health and development (Zar et al. 2015) provides details on the parent study). It is based on a peri-urban, low socio-economic neighbourhood in Paarl outside of Cape Town. Drakenstein is a stable community comprising approximately 200,000 people, and characterised by a high prevalence of health risk factors like depression, childhood trauma and poverty (Donald et al. 2019). It is considered representative of peri-urban regions across South Africa with most residents utilising primary public healthcare clinics.

### Population

Pregnant women between 20 and 28 weeks gestation were recruited from a public sector primary healthcare facility in the Drakenstein district, enrolled into the DCHS while attending routine antenatal care, and prospectively followed through childbirth and early childhood. This study included the subset of infants who were seen at the 14-week follow-up visit and had a 6-month developmental assessment.

### Measures

Demographic and clinical (physical and psychological) child measures were assessed. Variables included maternal social and biological risk and protective factors (including maternal education, employment, mental illness and substance use) and an infant neurodevelopmental assessment (Bayley Scales of Infant Toddler Development—BSID-III scale). Video-recorded dyadic interactions between mothers and infants were assessed for SP. Videos with at least 5 min unobstructed interactional visuals of infant and mother were deemed acceptable. Dyads were videotaped at 14-week well baby follow-up clinic visits.

### Video Recordings

At these 14-week visits, mothers were recorded interacting with their infants for 5–10 min. The video camera was positioned for a full-face view of infants and mothers in profile. A mirror adjacent to the infants’ seat included infants’ faces and whole bodies, as well as full-face reflections of mothers, in the frame. Mothers played freely with their infants without using toys.

### SP Paradigm

SP in parent–infant interactions—defined as “the parent and the child sharing positive affects in synchrony” (Puura et al. 2002) —was analysed from the first 5 min of the recording, a time span in which interrater reliability is good (Kemppinen et al. 2005). This had to be indicated by synchronised facial expressions like the mouth curving into a smile or laugh during gaze contact. If infants were sleepy or close to feeding, a second video was attempted when they were more alert. Tapes were observed at full speed, tagging all possible sequences of SP. Tagged parts were then reviewed at half speed, registering beginnings and endings of SP sequences per second. SP moments were measured by a single rater (AL) blinded to the history of the mother. Measurement for SP comprised three components: occurrence of an SP moment, total number of SP moments, and duration of the SP moment. AL received training in reliability by KP who developed this SP analysis method. To assess interrater reliability, 10% of the videos were randomly selected using the random number generator in Excel and were rated independently by two coders (AL and KP). The kappa values for the SP variables used in the analysis, i.e. the occurrence of SP sequences and the mean duration of SP (< 0.5 or > 0.5 s), were 0.79 and 0.46, respectively. The respective rates of interrater agreement were substantial for occurrence of SP moments and moderate for mean duration of SP157 (McHugh 2012).

### Child development: Bayley Scales of Infant and Toddler Development

The Bayley Scales of Infant and Toddler Development, Third Edition (BSID-III) (Bayley 2006) —a gold-standard observational measure of childhood development from 0 to 42 months —assessed child development at 24 months as part of the DCHS parent study. It has been validated and is considered culturally appropriate for South African application (Rademeyer and Jacklin 2013). It measures development by direct observation across five subscales: cognition, receptive and expressive language, and fine and gross motor development. For this study, the 24-month BSID-III composite cognitive, motor and language scores were generated using BSID-III normative and conversion tables, accounting for gestation at delivery. These scales were measured via direct observation by a trained physiotherapist blinded to the risk factors, and overseen by a paediatric neurologist (Donald et al. 2018). The original DCHS parent study analysis calculated raw and scaled scores using a normal US population, scaled to a mean of 10 and a standard deviation of 3. Poor developmental outcomes were assessed by categorising scores into ‘delay’ or ‘no delay’, defined by scoring ≤ 1 standard deviation below the mean scaled score (using a cut-off of 7). For the current study analysis, composite scores were used and categorised with a score < 85 indicating a ‘delay’ which was 1 SD from the mean for the South African population (le Roux et al. 2018).

## Maternal measures

### Several aspects of maternal mental health were assessed

The Edinburgh Postnatal Depression Rating Scale (EPDS) (Cox et al. 1987) is a 10-item self-report measure of recent depressive symptoms. Each item is scored on a frequency scaleranging from 0 to 3. The total score is obtained by summing individual item responses. A higher score indicates stronger depressive symptoms; a cut-off score ≥ 13 indicates probabledepression. Cronbach’s alpha (α) in the larger cohort of 1137 mothers = 0.79.

The WHO-endorsed Self-Reporting Questionnaire-20 (SRQ-20) (Beuenberg and Orley, 1994) measures psychological distress. Prolifically used locally and internationally, it maintains good reliability and face validity (Harpham et al. 2003) and consists of 20 items assessing non-psychotic symptoms. Individual items are summed to generate a total score. A cut-off score ≥ 8 can help sort participants into “high risk” versus “low risk” (Harpham et al. 2003). The measure demonstrated adequate reliability (α = 0.82).

The Intimate Partner Violence (IPV) Questionnaire is a 12 item inventory adapted from the WHO multi-country study (Jewkes 2002) and the Women’s Health Study in Zimbabwe (Shamu et al. 2011) to assess lifetime and recent (past 12 months) exposure to emotional, physical and sexual IPV. Categories are assessed across multiple items measuring frequency and number of acts. Scoring guidelines for the DCHS categorised participants as above or below threshold depending on responses of low (once), mid (more than once) or high (many times) frequency of exposure to violence. Emotional abuse subscale: α = 0.85; physical abuse subscale: α = 0.86 & sexual abuse subscale: α = 0.83. Studies in South Africa have used the adapted IPV including in high-risk HIV-positive samples (Donald et al. 2019; Jewkes et al. 2010).

The World Mental Health Life Events Questionnaire (LEQ) is a 17-item tool which assesses exposure to stressful life events during the previous 12 months. The questionnaire used in this study is based on the items used in the South African Stress and Health Study (Myer et al. 2008). A total score is obtained by summing the total number of life events reported during this time frame; higher scores indicate greater exposure to stressful life events, with α = 0.71.

The Alcohol, Smoking and Substance Involvement Screening Test (ASSIST) (WHO Assist Working Group 2002) was developed by the WHO to detect and manage substance use among people employing primary healthcare services. It assesses substance use and substance-related risk across diverse categories of substances and is widely used in South Africa (van der Westhuizen et al. 2016). Total scores are obtained for each substance by summing individual item responses, with a higher score indicative of greater risk for substance-related health problems. For alcohol use, scores of 0–10 indicated low risk, 11–26 moderate risk, and scores > 26 a participant’s high risk of experiencing severe problems resulting from their current pattern of use and likely dependence (Humeniuk et al. 2010). The alcohol use subscale demonstrated adequate reliability (α = 0.91).

### Statistical Analysis

A series of bivariate models were was conducted to test the link between preceding perinatal *maternal* and *infant* factors on and SP at 3 ½ months. SP was assessed in two metrics: (1) frequency of SP moments [SP Frequency] and (2) occurrence of SP moments (i.e. presence vs absence) [SP Occurrence]. Due to the overdispersion of SP frequency data, negative-binomial regression models were conducted to gauge the potential impact of maternal and infant factors on SP frequency. Logistic regression was used to model SP occurrence regarding maternal and infant factors. Further sensitivity analyses were run to assess whether findings differed according to additional SP metrics which included (3) aggregated duration of SP [SP Sum] or (4) average duration of SP [SP Short-Average], both using negative-binomial regression modelling.

Perinatal maternal mental health factors like perinatal depression (EPDS), lifetime and recent intimate partner violence (IPV), psychological distress (SRQ-20), stressful life events (LEQ) and alcohol use (ASSIST) were measured at antenatal and/or 6–10-week postnatal visits. The maximum score across perinatal visits was used to minimise the amount of missing maternal mental health data in bivariate analyses of each mental health factor and SP. Early infant development was assessed using several continuous composite scales taken at 2 years, including cognition, motor, language, socio-emotional and general adaptive behaviour, each assessed in relation to SP. Additional factors like maternal age at birth, marital status, employment status, education, HIV status, infant sex, gestational age and birth weight were investigated. All models assumed a significance level of 0.05, and all estimates were presented with 95% confidence intervals. Analyses were run using SAS (V9.4) and R (R Core Team 2021).

### Ethical Considerations

The DCHS study was approved by the Faculty of Health Sciences Research Ethics Committee, University of Cape Town (401/2009) and Western Cape Provincial Research Committee (2011RP45). An amendment for the inclusion of the Shared Pleasure Analysis was granted (16/12/2016). At enrollment, mothers gave voluntary, written, informed consent in their preferred language (English, Afrikaans or isiXhosa) and were reconsented annually for study involvement. Parents gave consent for children younger than 12 months as per South African National HREC guidelines that extend the parameters for informed consent by parents to include infants specifically under 12 months of age (Department of Health: RSA 2015). All data were anonymised using a study number to code for each mother–infant dyad.

## Results

### Maternal and infant characteristics

A sample of 291 out of a possible 296 videos of mother–infant dyads was suitable for assessing Shared Pleasure (SP). Infant sex distribution comprised 54% females and 46% males. Mothers were young (mean age = 27 years (SD 5.9)), mostly unmarried (84%) and largely had at least a secondary school level education (95%). Seventy-three percent of mothers were unemployed (see Table 1), and the majority scored below the threshold for depression (63%) as measured by the EPDS. Seventy-six percent indicated a low risk of psychological distress as measured by the SRQ-20 screen. Most participants (86%) reported few (< 5) stressful events in the past year.

**Table 1:** Maternal characteristics

Variable		*n (%)*
**Age at birth (years)**		27 (5.9) *
**Marital status**	Married	47 (16%)
	Single/cohabiting	243 (84%)
**Education**	Primary	190 (65%)
	Secondary	85 (29%)
	Tertiary	16 (6%)
**Employment**	Employed	48 (16%)
	Unemployed	243 (84%)
**HIV status**	Infected	69 (24%)
	Negative	222 (76%)
**Depression (EPDS)**	Below threshold (< 13)	179 (63%)
	Above threshold (≥ 13)	103 (37%)
**Psychological distress (SRQ-20)**	Low risk (< 8)	213 (76%)
	High Risk (≥ 8)	69 (24%)
**Stressful life events (LEQ)**	< 5 events	242 (86%)
	≥ 5 events	39 (14%)
**Intimate partner violence (IPV)**		
Lifetime (exposure > 12 months)		
	Above threshold	154 (54%)
	Below threshold	131 (46%)

Table 1 reports on maternal characteristics and mental health measures.

Table 2 reports on infant characteristics and infant BSID-III composite scores corrected for gestation.

**Table 2:** *Infant characteristics*

Variable	*Mean (sd)*
**Sex**	
Female n (%)	136 (46%)
Male n (%)	155 (54%)
**Gestational age (weeks)**	38.5 (2.7)
**Birth weight (kg)**	3.1 (0.6)
**BSID-111 composite scores**	
**Motor**	91.8 (12.1)
**Cognition**	85.3 (10)
**Language**	83.9 (11.8)
**Socio-emotional**	115.5 (20.1)
**Adaptive behaviour**	82.1 (12.2)
Recent (exposure ≤ 12 months)	
Above threshold	113 (45%)
Below threshold	140 (55%)
**Risk of alcohol use disorder (ASSIST)**	
Low (< 10)	229 (81%)
Moderate (11–26)	35 (12%)
High (> 26)	19 (6.7%)

*Note:* *mean (sd)

### SP moments and their associated factors

**Fig. 1:**
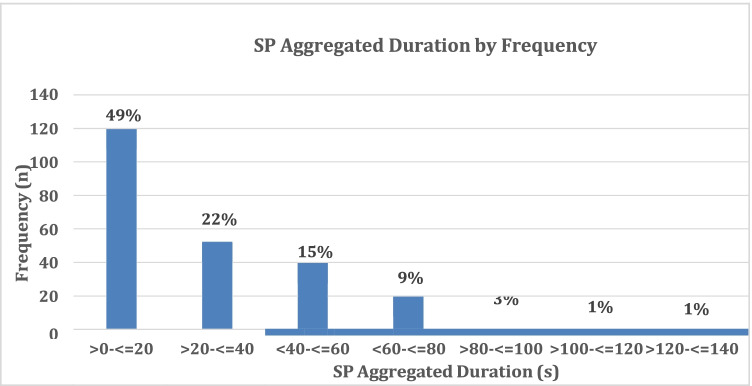
Aggregated Frequency and Duration of SP moments

Of the total sample (N = 291), 239 (82%) maternal–infant dyads experienced SP moments [SP occurrence], displaying a median of 5 (IQR: 1–11) SP moments [SP frequency]. The maximum number of SP moments recorded in a single dyad was 31, with 50% of dyads having ≤ 5 moments. The minimum duration of an SP moment was 0.5 s and the maximum 28 s. The maximum aggregated duration for all SP moments was 133 s. The median aggregated duration of SP moments [SP Sum] was 16 s (IQR: 3–36 s), with a median SP short-average of 3 s (IQR: 2–4 s). The aggregated duration and frequency of SP moments are presented in Fig. 1.

### Mental health and other maternal factors

Maternal age at birth, employment status, education, marital status and HIV status were associated with neither SP occurrence nor SP frequency (see Online Resource 1).

Unexpectedly, perinatal maternal depression was associated with a significantly greater number of shared positive moments (SP frequency) (*ICC* = 1.03[1.00–1.06], *p* = 0.040). However, SP frequency appeared to be unrelated to EPDS among depressed mothers (n = 103) (*ICC* = 0.98[0.78–1.27], *p* = 0.893) and rather driven by mothers who were not depressed (n = 179) (*ICC* = 1.42[1.13–1.76], *p* = 0.001). These findings were not further supported when looking at SP occurrence as the outcome (*p* > 0.05), and may then potentially be viewed as coincidental, especially given the absence of clinician-evaluated delineation between mothers who were clinically depressed relative to those who weren’t.

There were no significant relationships between other perinatal maternal mental health factors and SP frequency or occurrence, including lifelong or recent exposure on the IPV, stressful life events (LEQ), psychological distress (SRQ-20) and risk of Alcohol Use Disorder (ASSIST).

### Infant birth and development

Greater gestational age (*OR* = 1.45[1.10–1.93], *p* = 0.008) and birth weight (*OR* = 1.50[1.12- 2.02], *p* = 0.006) were significantly associated with increased odds of SP occurrence. Furthermore, none of the infant development scales were associated with SP frequency or occurrence at age 24 months (see Online Resource 2).

### Sensitivity analysis findings

Sensitivity analyses confirmed similar findings to those from main analyses (see Online Resource 3), greater gestational and birth ages significantly linked with a higher average SP duration, whereas greater risk for alcohol use disorder was significantly associated with a lower SP short-average duration [SP Short-average] (*IRR* = 0.90[0.76–1.06], *p* = 0.027). However, a sub-analysis limited to mothers at risk for alcohol use disorder (n = 54) appeared to dismiss this relationship (*IRR* = 0.97[0.76–1.22], *p* = 0.768), likely reflecting an underpowered analysis.

## Discussion

This study demonstrated 1) a high occurrence of positive interactional affectivity in a general population sample, 2) SP moments associated with higher age of gestation at delivery and infant birth weight, and 3) SP moments unrelated to maternal mental health or infant developmental outcomes in this cohort.

SP moments in this sample of 3½-month-old infant-and-mother pairs were common (82%). Transpiring SP moments were enduring (median aggregated length of 17 s) and frequent, similar to previous SP studies (Latva et al. 2013; Puura et al. 2013). SP occurred in only 20.5% of dyads at 2 months (Lachman et al. 2021), but at 3 months infants typically took to their developmental milestones and spent more time smiling, specifically when gazing at their mothers (Messinger et al. 2001; Yale et al. 2003). So despite significant environmental stressors in our context (e.g. 84% unemployment rate), mothers could respond sensitively to and engage positively with their infants. The presence of SP moments was significantly associated with an older gestation age and higher birth weight at delivery. Previous SP studies have not fully explored these associations (Latva et al. 2013; Mäntymaa et al. 2015) and further work is needed to investigate the mechanisms which underlie these associations. The finding in our sample may be related to the neurotypical behaviour of full-term or closer- to-term infants during face-to-face interactions, characterised by typically “wide” smiles (Segal et al. 1995). An older (closer-to-term) gestational age may also foretell a better developed capacity for pro-social engagement in the first 2 years of life (Dueker et al. 2016). Underlying infant reactivity and self-regulation can furthermore affect infant ability to signal or seek out interaction with caregivers and depend on both temperamental vulnerabilites and caregiver sensitivity(Rothbart and Bates 2006).

Contrary to our hypothesis, SP moments were not associated with better maternal mental health or infant developmental outcomes in this cohort. Our findings contradict other studies linking maternal stressors and depressive symptoms with lower SP (Puura et al. 2019; Lachman et al. 2021). Although mothers with depressive symptoms can interact positively with their infants (Goodman and Gotlib 1999; Cornish et al. 2008). In prior work in the South African context, we found that SP was lower in mothers with severe mental illness (Lachman et al.^2021^). There are very few opportunities and tools available to sensitively and appropriately assess infant and maternal mental health in vulnerable populations, especially those in LMICs (Keller et al., 2018). Further work is needed to determine whether SP may be a culturally appropriate screen for mother–infant connectedness. It may therefore be a combination of risks rather than depression alone that determines adverse outcomes (Cornish et al. 2008). Increasingly, literature emphasises that even under adverse conditions, significant resilience may be evident (Ellis et al. 2017). Thus, for example mothers in this cohort also demonstrate remarkable resilience. Further work is, however, needed to assess SP in mothers with more severe mental illness.

Several limitations deserve emphasis. Firstly, this study did not consider a number of factors relevant to shared pleasure, including individual factors like maternal sensitivity and infant temperament. Secondly, it failed to measure a number of variables that impact synchronicity. Thirdly, the assessment of video content did not allow for implementation of computerised analysis methods and only moderate interrater agreement for mean duration of SP was found.

Taken together, our work indicates that despite mother–infant dyads facing risk factors for suboptimal development, there is frequent occurrence of positive SP moments in reciprocal dyadic interactions. Significant disruption in shared pleasure may be present only in extreme cases (e.g. mothers with severe mental disorders). Further work is needed to investigate the mechanisms underlying the associations between early mother-infant synchronicity and better outcomes noted here, and to assess whether SP may serve as a culturally appropriate screen for assessing connectedness.

## Supplementary Information

Below is the link to the electronic supplementary material.

Supplementary file1 (DOCX 20 KB)
