# Supplementary material for: The Shared Pleasure Paradigm: A study in an observational birth cohort in South Africa
Source: Arch Womens Ment Health. 2022 Jan 5;25(1):227–35. doi: 10.1007/s00737-021-01199-0 (PMC8784495; doi:10.1007/s00737-021-01199-0)
Supplement: Supplementary file 1 — Supplementary file1 (DOCX 20 KB) [file 737_2021_1199_MOESM1_ESM.docx]

| **Appendicies**  **Supplementary table 1** |  |  |
| --- | --- | --- |
| *Bivariate models: Maternal factors and SP frequency and occurrence* | | |
| Models | *Estimate [CI]* | *p-value* |
| *Outcome: SP frequency^1^* |  |  |
| **Maternal age at birth** | 0.98 [0.87-1.12] | 0.807 |
| **Marital status** Single/co-habiting | 1.12 [0.79-1.58] | 0.506 |
| **Education** Secondary | 0.90 [0.68-1.20] | 0.475 |
| Teritary | 1.17 [0.69-2.12] | 0.587 |
| **Employment**  Employed | 0.88 [0.63-1.26] | 0.473 |
| **HIV status** Infected | 1.24 [0.93-1.68] | 0.153 |
| **Depression (EPDS)** | 1.03 [1.00-1.06] | 0.040* |
| **Psychological distress (SRQ-20)** | 1.01 [0.86-1.15] | 0.859 |
| **Stressful life events (LEQ)** | 0.99 [0.88-1.12] | 0.865 |
| **Intimate partner violence (IPV)** |  |  |
| Lifetime (exposure >12 months) | 0.88 [0.68-1.14] | 0.344 |
| Recent (exposure ≤ 12 months) | 0.94 [0.72-1.23] | 0.663 |
| **Risk of alcohol use disorder (ASSIST)** | 0.95 [0.84-1.08] | 0.439 |
| *Outcome: SP occurrence^2^* |  |  |
| **Maternal age at birth** | 1.15 [0.85-1.58] | 0.372 |
| **Marital status** Single/co-habiting | 0.77 [0.30-1.74] | 0.554 |
| **Education** Secondary | 1.19 [0.61-2.40] | 0.623 |
| Teritary | 1.64 [0.43-10.71] | 0.527 |
| **Employment**  Employed | 0.68 [0.33-1.50] | 0.320 |
| **HIV status** Infected | 1.04 [0.53-2.20] | 0.906 |
| **Depression (EPDS)** | 1.04 [0.97-1.10] | 0.255 |
| **Psychological distress (SRQ-20)** | 1.03 [0.76-1.42] | 0.837 |
| **Stressful life events (LEQ)** | 0.97 [0.72-1.33] | 0.820 |
| **Intimate partner violence (IPV)** |  |  |
| Lifetime (exposure >12 months) | 0.80 [0.43-1.48] | 0.487 |
| Recent (exposure ≤ 12 months) | 0.69 [0.35-1.34] | 0.272 |
| **Risk of alcohol use disorder (ASSIST)** | 0.77 [0.59-1.02] | 0.055 |

*Note:1 = Incidence rate ratios; 2 = Odds ratio; * p <0.05*

| **Supplementary table 2** | | |
| --- | --- | --- |
| *Bivariate models: Child factors and SP frequency and occurrence* | | |
| Models | *Estimate [CI]* | *p-value* |
| *Outcome: SP frequency^1^* |  |  |
| **Sex** Male | 1.10 [0.85-1.41] | 0.478 |
| **Gestational age (in weeks)** | 1.03 [0.89-1.18] | 0.649 |
| **Birth weight (kg)** | 1.11 [0.96-1.28] | 0.105 |
| **BSID-111 composite scores** |  |  |
| **Motor** | 0.92 [0.80-1.05] | 0.245 |
| **Cognition** | 0.96 [0.83-1.11] | 0.581 |
| **Language** | 0.96 [0.83-1.11] | 0.562 |
| **Socio-emotional** | 0.92 [0.80-1.06] | 0.233 |
| **Adaptive behaviour** | 0.98 [0.85-1.12] | 0.743 |
| *Outcome: SP Occurrence^2^* |  |  |
| **Sex** Male | 1.42 [0.78-2.60] | 0.258 |
| **Gestational age (in weeks)** | 1.45 [1.10-1.93] | 0.008* |
| **Birth weight (kg)** | 1.50 [1.12-2.02] | 0.006* |
| **BSID-111 composite scores** |  |  |
| **Motor** | 1.20 [0.84-1.75] | 0.329 |
| **Cognition** | 1.16 [0.82-1.61] | 0.402 |
| **Language** | 0.68 [0.46-1.00] | 0.054 |
| **Socio-emotional** | 1.16 [0.81-1.64] | 0.415 |
| **Adaptive behaviour** | 1.21 [0.85-1.73] | 0.298 |

*Note:1 = Incidence rate ratios; 2 = Odds ratio; * p <0.05*

| **Supplementary table 3** |  |  |
| --- | --- | --- |
| *Sensivity analyses: Bivariate models* | | |
| Models | *Estimate [CI]* | *p-value* |
| ***Maternal factors*** |  |  |
| *Outcome: SP sum^1^* |  |  |
| **Maternal age at birth** | 1.00 [0.85-1.17] | 0.957 |
| **Marital status** Single/co-habiting | 1.31 [0.83-1.98] | 0.218 |
| **Education** Secondary | 0.89 [0.63-1.27] | 0.510 |
| Teritary | 1.22 [0.64-2.65] | 0.579 |
| **Employment**  Employed | 0.77 [0.51-1.20] | 0.223 |
| **HIV status** Infected | 1.29 [0.90-1.89] | 0.176 |
| **Depression (EPDS)** | 1.02 [0.99-1.06] | 0.165 |
| **Psychological distress (SRQ-20)** | 1.00 [0.86-1.18] | 0.994 |
| **Stressful life events (LEQ)** | 0.98 [0.85-1.14] | 0.766 |
| **Intimate partner violence (IPV)** |  |  |
| Lifetime (exposure >12 months) | 0.92 [0.67-1.27] | 0.614 |
| Recent (exposure ≤ 12 months) | 0.89 [0.64-1.24] | 0.491 |
| **Risk of alcohol use disorder (ASSIST)** | 0.95 [0.82-1.12] | 0.534 |
| *Outcome: SP short-average^1^* |  |  |
| **Maternal age at birth** | 1.03 [0.95-1.12] | 0.461 |
| **Marital status** Single/co-habiting | 1.15 [0.92-1.45] | 0.221 |
| **Education** Secondary | 1.01 [0.84-1.20] | 0.926 |
| Teritary | 1.16 [0.82-1.62] | 0.394 |
| **Employment**  Employed | 0.82 [0.66-1.03] | 0.091 |
| **HIV status** Infected | 1.15 [0.96-1.38] | 0.129 |
| **Depression (EPDS)** | 1.01 [0.99-1.02] | 0.308 |
| **Psychological distress (SRQ-20)** | 1.01 [0.93-1.10] | 0.794 |
| **Stressful life events (LEQ)** | 0.98 [0.90-1.06] | 0.576 |
| **Intimate partner violence (IPV)** |  |  |
| Lifetime (exposure >12 months) | 0.95 [0.80-1.11] | 0.504 |
| Recent (exposure ≤ 12 months) | 0.90 [0.76-1.06] | 0.191 |
| **Risk of alcohol use disorder (ASSIST)** | 0.91 [0.83-0.99] | 0.027* |
| ***Infant factors*** |  |  |
| *Outcome: SP sum^1^* |  |  |
| **Sex** Male | 0.99 [0.72-1.36] | 0.962 |
| **Gestational age (in weeks)** | 1.06 [0.88-1.25] | 0.503 |
| **Birth weight (kg)** | 1.16 [0.97-1.39] | 0.059 |
| **BSID-111 composite scores** |  |  |
| **Motor** | 0.93 [0.79-1.11] | 0.431 |
| **Cognition** | 0.98 [0.80-1.18] | 0.807 |
| **Language** | 1.00 [0.83-1.20] | 0.998 |
| **Socio-emotional** | 0.93 [0.78-1.11] | 0.413 |
| **Adaptive behaviour** | 1.01 [0.84-1.21] | 0.907 |
| *Outcome: SP short-average^1^* |  |  |
| **Sex** Male | 1.04 [0.89-1.23] | 0.592 |
| **Gestational age (in weeks)** | 1.11 [1.02-1.21] | 0.015* |
| **Birth weight (kg)** | 1.12 [1.03-1.22] | 0.006* |
| **BSID-111 composite scores** |  |  |
| **Motor** | 1.05 [0.95-1.15] | 0.345 |
| **Cognition** | 1.02 [0.93-1.12] | 0.740 |
| **Language** | 0.96 [0.87-1.05] | 0.336 |
| **Socio-emotional** | 1.00 [0.91-1.10] | 0.995 |
| **Adaptive behaviour** | 1.03 [0.94-1.13] | 0.567 |

*Note:1 = Incidence rate ratios; 2 = Odds ratio; * p <0.05*
